# Learning Curve for Metastatic Liver Tumor Open Resection in Patients with Primary Colorectal Cancer: Use of the Cumulative Sum Method

**DOI:** 10.3390/ijerph19031068

**Published:** 2022-01-19

**Authors:** Bartlomiej Banas, Piotr Gwizdak, Paulina Zabielska, Piotr Kolodziejczyk, Piotr Richter

**Affiliations:** 1 First Department of Surgery, Jagiellonian University Medical College, 30-688 Kraków, Poland; piotr0gwizdak@gmail.com (P.G.); piotr.1.kolodziejczyk@uj.edu.pl (P.K.); piotr.richter@uj.edu.pl (P.R.); 2 Sub-Department of Social Medicine and Public Health, Department of Social Medicine, Pomeranian Medical University, 71-210 Szczecin, Poland; paulina.zabielska@pum.edu.pl

**Keywords:** learning curve, major liver resections, metastatic liver tumor, small liver resections

## Abstract

Background: Liver resections have become the first-line treatment for primary and metastatic tumors and, therefore, are considered a core aspect of surgical training. This study aims to evaluate the learning curve of the extent and safety of liver resection procedures for patients with metastatic colorectal cancer. Methods: This single tertiary center retrospective analysis includes 158 consecutive cases of small liver resection (SLR) (n = 107) and major liver resection (MLR) (n = 58) procedures. A cumulative sum control chart (CUSUM) method was used to investigate the learning curve. Results: The operative time, total blood loss level, and incidence of adverse effects showed a learning curve. For SLRs, the CUSUM curve for operative time and blood loss level peaked at the 19th and 17th case, respectively, while for MLRs, these curves peaked at the 28th and 24th case, respectively. The CUSUM curve for minor adverse effects (MAEs) and severe adverse effects (SAEs) showed a downward slope after the 16th and 68th procedures in the SLRs group and after the 29th and 39th procedures in the MLRs cohort; however, it remained within the acceptable range throughout the entire study. Conclusion: SLR procedures were performed faster with less intraoperative blood loss and shorter postoperative stays than MLRs, and a higher number of completed procedures was required to gain stabilization and repeatability in the operating time and intraoperative blood loss level. In MLR procedures, the reduction of SAEs was accomplished significantly later than the stabilization of the operative time and intraoperative blood loss level.

## 1. Introduction

The liver is a critical organ and a common metastatic site of many malignancies, including breast, colorectal, lung, ovarian, and gastric cancers, gastrointestinal stromal tumors (GISTs), and melanomas [1,2,3,4,5,6,7,8]. These metastases can be recognized synchronously with primary tumor diagnoses or can occur up to decades after the primary radical treatment completion. Colorectal cancer has a high liver metastatic potential. The proportion of patients with synchronous liver metastases diagnosed with primary tumors varies from 14.5% to 26.5%, and patients are more often diagnosed with left-sided than right-sided colon cancer [1,2,9]. In a five-year follow-up study, the incidence of liver metastases in colorectal cancer survivors increased up to 30% [2,9]. Surgical treatment is a first-choice therapy for liver metastases; therefore, every surgeon, including both general surgery and oncologic surgery specialists, should be able to effectively manage patients with primary and secondary liver tumors.

A surgeon’s necessary skills and experience acquisition can be evaluated utilizing established learning curves. Using this method, the minimum number of procedures required to reach the same intra- and postoperative outcomes by a surgeon applying a given technique can be predicted. The skills acquisition progression can be presented graphically, helping to identify the point of improvement [10].

To identify inflection points corresponding to the learning curve, a cumulative sum analysis (CUSUM) may be used [11]. Originally, this technique was primarily employed for monitoring performance and detecting areas for improvement in the industrial sector; thereafter, in the late 1970s, it was adopted for analyzing learning curves first for surgical procedures and then for various other medical methods [12,13].

Since then, many studies have evaluated learning curves in liver surgery. These studies mostly focused on primary tumors and compared different surgical approaches, including minimally invasive techniques such as laparoscopic liver resection, robotic surgery, and hepatectomies for liver transplantations [14,15,16,17]. However, the balance between the learning curve and length of postoperative hospital stay as well as minor adverse effects (MAEs) and severe adverse effects (SAEs) has never been specifically investigated. The primary aim of this study was to define a standard educational path for evaluating the learning curve based on the operation time and intraoperative blood loss level in patients with colorectal cancer. The secondary endpoint analyzed was patients’ length of postoperative hospital stay and occurrence of MAEs and SAEs in the context of a learning curve.

## 2. Materials and Methods

### 2.1. Patient and Procedures Characteristics

A retrospective analysis of the medical registry comprising patients who underwent laparotomy and liver tumor resection due to metastases was approved by the Institutional Review Board. The study covered the period from 01 January 2010 until 31 December 2015, and all the patients received surgery performed by the same surgeon—a specialist in general surgery and in training in hepatobiliary surgery. The inclusion criteria were as follows: (1) aged 18 years or above, (2) liver metastases of colorectal cancer confirmed in histopathological reports, and (3) no previous liver surgeries; cases with missing data were excluded. The endpoints analyzed were as follows: (1) operating time measured from skin incision to skin closure; (2) intraoperative blood loss level, defined as blood volume removed by suction; and (3) postoperative hospital stay length from the first postoperative day to hospital discharge date. Procedures including left or right were considered major liver resections (MLRs). Additionally, two cases of formal posterior segments 6 and 7 resections were included in the MLRs group, because they presented outliers in operating time and intraoperative blood loss if considered small liver resections (SLRs). Nonanatomical tumor excisions and segments 1, 2, 3, 4b, 5, and 8 resections were described as SLRs. Patient safety was evaluated based on the presence of adverse events, an inevitable aspect of the medical services provided, and these events were defined as MAEs and SAEs. MAEs included: (1) wound infection; (2) prolonged hospital stay (>10 days); and (3) hematoma managed nonsurgically. SAEs were as follows: (1) patient death; (2) admission to the intensive care unit; (3) reoperation due to intraperitoneal bleeding; (4) wound dehiscence; (5) hepatobiliary fistula; and (6) blood and/or frozen plasma transfusions. Only two cases of posthepatectomy liver failure (PHLF)—as defined per the International Study Group of Liver Surgeries (ISGLS) consensus—were reported in the MLRs group, therefore, they were withdrawn for further analysis [18].

### 2.2. Statistical Analysis

A cumulative sum control chart (CUSUM) analysis was used to investigate the learning curve of SLRs and MLRs separately in terms of operative time as well as intraoperative blood loss level and the length of hospital stay [12,13,19]. The cumulative difference between the values of each patient’s variables and the mean variable values was plotted with the patients arranged chronologically. The change point identified in the CUSUM curve was the point that divided SLRs and MLRs into early and late groups based on the operative time (time early: tE; time late: tL), the intraoperative blood loss level (blood loss early: blE, and blood loss late: blL), and the length of postoperative hospital stay (postoperative hospital stay early: hE, and postoperative hospital stay late: hL). Body mass index (BMI) was calculated by dividing the body mass by the square of the body height and is presented as kg/m2. Data are presented as the mean ± standard deviation (SD) or as the median ± standard error of the mean (SEM) depending on their distributions, which were checked using the Kologomorov–Smirnov test. Then, the study groups were compared using the parametric Student’s t-test and the nonparametric Mann–Whitney U test as appropriate. For evaluating categorical data, the chi-squared test was employed. A p value of 0.05 was considered statistically significant, and all the calculations were performed using STATISTICA data analysis software, (TIBCO Software Inc. 2017, version 13.0, Palo Alto, CA, USA).

## 3. Results

### 3.1. Patients and Procedure

From the total number of 169 identified cases, 4 were excluded due to missing data, and in 7, metastases were not confirmed in the final pathological report. Accordingly, 158 cases were analyzed. The median time from primary colorectal surgery was 45 months (IQR: 36). A total of 107 cases (67.73%) were classified as SLRs and 51 (32.27%) as MLRs based on the criteria described in the Methodology section. The study group comprised 76 (49.10%) males and 82 (51.90%) females of a mean age of 57.60 years (±13.03) and a mean BMI of 26.88 kg/m^2^ (±4.57). Detailed characteristics of the patients and procedures are presented in Table 1.

### 3.2. Learning Curve Endpoints

**Figure 1:**
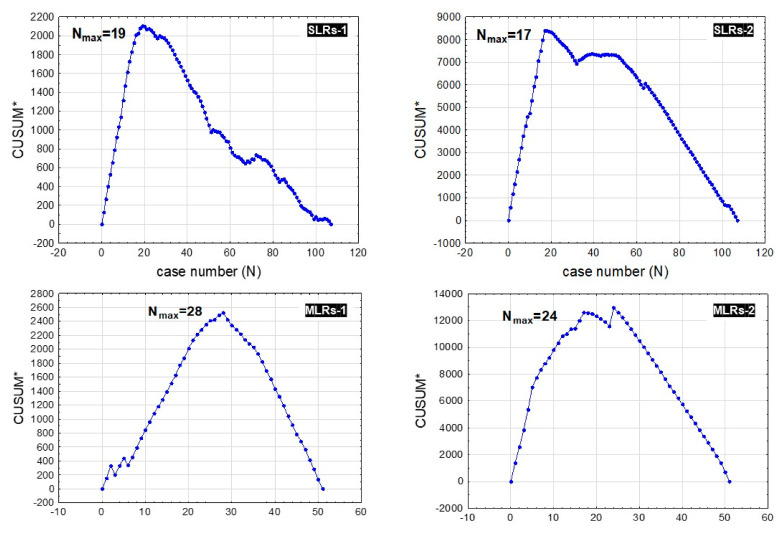
Cumulative sum control chart of operative (CUSUM *) time (SLRs-1) and intraoperative blood loss (SMLRs-2) against the number of patients with small liver resections (SMLs), and CUSUM time (MLRs-1) and intraoperative blood loss (MLRs-2) against number of patients with major liver resections (MLRs); N = 107.

The median operating time in the 158 consecutive cases was 205 min (IQR: 33) and was significantly shorter in SLR procedures than in MLRs (see Table 1). The median intraoperative blood loss level in the whole cohort was 330 mL (IQR: 45), and similar to the operating time, it was significantly lower in the SLRs group than in the MLRs group (see Table 1). In SLR procedures, based on operating time, CUSUM analysis identified procedure no. 19 as the cut-off point of gaining a stable and repeatable surgical experience; in MLRs, this point was established at procedure no. 28 (Figure 1).

Subsequent analysis showed that in the tE-SLRs group (defined as procedures no. 1–19), the median operating time was longer [310 min. (IQR: 40) vs. 135 min. (IQR: 35); *p* < 0.001]; similarly, the median intraoperative blood loss level was higher [750 mL. (IQR: 150) vs. 150 mL. (IQR: 118); *p* < 0.001], and the median postoperative hospital stay length was longer [7 days (IQR: 4) vs. 5 days (IQR: 2); *p* = 0.001] compared to those observed in tL-SLRs procedures (i.e., 20–107), and the differences were significant. No differences in patients’ median age and BMI were observed between the analyzed tE-SLRs and tL-SLRs groups [54.58 years ± 14.90 vs. 58.98 years ±13.62; *p* = 0.565 and 27.27 kg/m^2^ ± 4.49 vs. 26.69 kg/m^2^ ± 4.65; *p* = 0.909].

In the MLR group, based on the learning curve and CUSUM results, procedures no. 1–28 were classified as tE-MLRs, while cases no. 29–51 were classified as tL-MLRs. No differences in mean patient age or mean BMI were observed between the tE-MLR and tL-MLR procedures [57.84 years ±13.41 vs. 55.47 years ± 9.47; *p* = 0.463 and 25.74 kg/m^2^ ± 3.33 vs. 27.50 kg/m^2^ ± 4.80; *p* = 0.286]. In the tE-MLR group, compared with the tL-MLR cohort, we observed a significantly longer operative time [460 min. (IQR: 25) vs. 285 min. (IQR: 67.5); *p* < 0.001] and an increased intraoperative blood loss level [1400 mL (IQR: 800) vs. 352 mL (IQR: 37.5); *p* < 0.001] with a prolonged postoperative hospital stay length [12 days (IQR: 37) vs. 6.5 days (IQR: 3); *p* = 0.003], and these differences were significant.

When taking into consideration intraoperative blood loss level cut-off points for the learning curve, procedure no. 17 for SLRs and no. 24 for MLRs allowed us to distinguish tE-MLRs (no. 1–17) and tL-MLRs (no. 18–107) (Figure 1). No significant differences in mean patient age or mean BMI were observed between blE-SLRs and blL-SLRs [53.35 years ± 15.31 vs. 59.11 years ± 13.51; *p* = 0.447 and 26.81 kg/m^2^ ± 4.47 vs. 26.79 kg/m^2^ ± 4.66; *p* = 0.992]. In the blE-SLRs group, however, we observed significantly longer operative times [310 min. (IQR: 30) vs. 165 min. (IQR: 35); *p* < 0.001], increased intraoperative blood loss levels [800 mL (IQR:140) vs. 152.5 mL (IQR: 115); *p* < 0.001], and prolonged postoperative hospital stays [7 days (IQR: 4) vs. 5 days (IQR: 2); *p* = 0.002] compared with those observed in the blL-SLRs cohort.

Again, based on the learning curve and CUSUM results in the MLRs group, procedures no. 1–24 were classified as blE-MLRs, while cases no. 25–51 were classified as blL-MLRs, and no significant differences in mean patient age or mean BMI were observed between blE-MLRs and blL-MLRs [55.68 years ± 13.34 vs. 57.01 years ± 8.64; *p* = 0.674 and 27.23 kg/m^2^ ± 4.72 vs. 26.99 kg/m^2^ ± 4.99; *p* = 0.870]. A significantly longer operative time [455 min. (IQR: 30) vs. 267 min. (IQR: 125); *p* < 0.001], an increased intraoperative blood loss level [1320 mL (IQR: 700) vs. 340 mL (IQR: 45); *p* < 0.001], and a prolonged postoperative hospital stay [10 days (IQR: 18) vs. 7 days (IQR: 3); *p* = 0.031] were observed in the blE-MLRs cohort compared with those in the blL-MLRs group.

### 3.3. Patient Safety

In the entire SLR cohort, 22 (20.56%) minor and 15 (14.08%) severe adverse effects occurred, including: for MAE, (1) wound infection—5; (2) prolonged hospital stay (>10 days)—11; (3) nonsurgically managed hematoma—8; and for SAE, (1) reoperation due to intraperitoneal bleeding—4; (2) wound dehiscence3; and (3) blood and/or frozen plasma transfusions—11. In the tE-SLR group, both MAEs and SAEs were observed more frequently than in the tL-SLRs cohort [10/19 (52.63%) vs. 12/88 (13.63%); *p* < 0.001 and 6/19 (31.57%) vs. 9/88 (10.23%); *p* = 0.002, respectively]. Similarly, both MAEs and SAEs were observed more frequently in blE-SLRs than in blL-SLRs procedures [9/17 (52.94%) vs. 13/90 (14.44%); *p* < 0.008 and 6/17 (35.29%) vs. 9/90 (10.00%); *p* = 0.025].

In the MLR cohort, 25 (49.02%) MAEs and 13 (25.49%) SAEs occurred. MAEs included: (1) wound infection—6; (2) prolonged hospital stay (>10 days)—14; and (3) nonsurgically managed hematoma—6. SAEs were as follows: (1) patient death—1; (2) admission to intensive care unit—2; (3) reoperation due to intraperitoneal bleeding—2; (4) hepatobiliary fistula—2; and (6) blood and/or frozen plasma transfusions—5.

**Figure 2:**
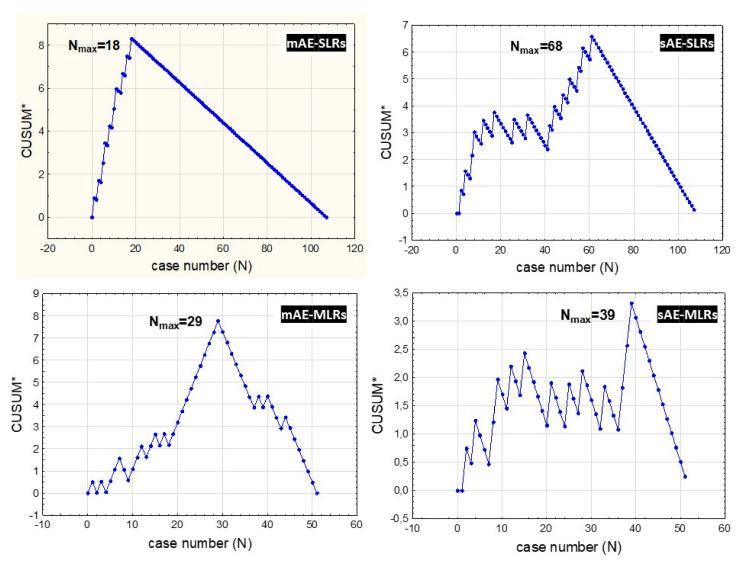
Cumulative sum control chart of operative (CUSUM *) minor adverse effects (MAEs) and severe adverse effects (SAEs) against the number of patients with small liver resections (SMLs) and CUSUM MAEs and SAEs against the number of patients with major liver resections (MLRs); N = 107.

In the tE-MLR group, both MAEs and SAEs were observed more frequently than in the tL-MLR cohort [21/28 (75.00%) vs. 4/23 (17.39%); *p* = 0.013 and 10/28 (32.14%) vs. 3/23 (26.08%); *p* = 0.149, respectively]; however, the latter was statistically insignificant. Both MAEs and SAEs were observed more frequently in blE-MLRs than in blL-MLRs: 18/28 (64.29%) vs. 7/23 (30.43%); *p* = 0.152 and 8/28 (28.57%) vs. 5/23 (21.74 %); *p* = 0.667], but the differences were insignificant. Furthermore, the CUSUM analysis of MAEs and SAEs showed that in the SLRs cohort, the incidence of MAEs decreased after the 19th procedure, which was consistent with the operative time and intraoperative blood loss level; however, a stable decrease in SAEs was accomplished after the 61st procedure, which was significantly higher than the operative time and intraoperative blood loss peak points. In the MLR group, the MAE stabilized after the 29th procedure, and stabilization of SAEs was observed after the 39th procedure, significantly later than operative time and intraoperative blood loss level equilibrium point (Figure 2).

## 4. Discussion

In this study, we demonstrated different learning curves for SLRs and MLRs procedures based on operating time and intraoperative blood loss analysis. Although simultaneous resections of segments 6 and 7 are not routinely classified as MLRs, according to our experience, these procedures require extended operating time and result in higher blood loss compared to others bisegmentomies. A similar classification was also presented in the 2008 Louisville Statement that defined MLRs as hemihepatectomies, trisectionectomies, and resection of the difficult posterior segments (that is, segments 4a, 6, and 7) which should be practiced in high-volume centers [20]. Additionally, our classification of liver resection is consistent with Chan et al.’s study which analyzed the learning curve for major hepatectomy in 151 patients who underwent 156 laparoscopic liver resections, thus making our results comparable with other studies [14].

As expected, fewer procedures are required to gain operating time and intraoperative blood loss level stabilization for SLRs than for MLRs. Additionally, SLR procedures exhibited shorter operating times and lower intraoperative blood loss levels than MLRs. These results are similar to Chan et al.’s findings, who reported shorter operating times, lower blood loss levels, and shorter hospital stay lengths for minor hepatectomies than for major hepatectomies [14]. However, it must be emphasized that our results cannot be directly compared with Chan et al.’s, who used a laparoscopic approach, as we performed open liver resections. Additionally, in that study, the learning curve analysis was applied only to major hepatectomies, identifying procedure no. 25 as a cut-off point distinguishing early from late procedures, which is comparable with our conclusions. In contrast to the above results, Nomi et al. analyzed 173 patients undergoing major hepatectomy procedures and, based on operating time, identified a three-phase learning curve with an initial stage comprising 45 cases [15]. In this study, however, the laparoscopic approach was analyzed, and a heterogeneous group of patients comprised cases of malignant and benign tumors as well as primary and metastatic liver cancers; thus, these results cannot be directly related to our conclusions [15]. Comparing laparoscopy versus laparotomy in small liver resections, Morino et al. confirmed a longer operating time but lower blood loss level in the laparoscopic group [21]. However, the mortality and morbidity rates in the two groups were comparable [21]. Similarly, Memeo et al., who analyzed 45 patients treated with laparoscopic liver resection and matched them with open liver resection cases, concluded that the laparoscopic approach resulted in shorter operative times, better resection margins, lower postoperative complications, and shorter hospital stays [22]. Similarly, de Sandro et al. indicated that, for patients with hepatocellular carcinoma, minor laparoscopic liver resection is a safe and feasible procedure, especially for cases complicated with liver cirrhosis, with fewer postoperative complications, lower operative blood loss levels, and shorter hospital stays along with comparable survival and recurrence-free survival rates [23]. The largest cohort of 782 hepatectomies was analyzed by Vigano et al., who found that liver resection with a laparoscopic approach is a safe operating technique and feasible for both primary and metastatic liver malignancies [24].

However, the novelty of the present study is a detailed analysis of patient safety based on MAE and SAE incidences using CUSUM testing. It has demonstrated that the most often analyzed indicators such as operating times and intraoperative blood loss levels are not simple surrogates for patient safety, especially in major liver resection surgeries. Our study shows that operation times and intraoperative blood loss levels stabilize significantly earlier, along with complications risk reduction and patient safety improvement. According to our analysis of MLR procedures, operative time improvement is gained after 28 procedures, while intraoperative blood loss is reduced even earlier—after completing 24 operations. Nevertheless, a significant reduction in SAEs in the MLR group was achieved only after as many as 39 operations. In the SLRs group, this was observed after even more—69 procedures. This can be explained by the two-fold larger SLRs group and the lower incidence of SAEs in this cohort than in the MLR cohort.

The main strength of this study is the large number of consecutive cases, especially SLRs. Additionally, separate analyses performed for SLR and MLR procedures provide new insight into the process of acquiring surgical skills and experience. We are, however, aware that the present series has several limitations. Firstly, this report was based on a single surgeon’s experience with established surgical techniques and did not include different operators. Secondly, we recognize that a retrospective analysis may be biased due to unknown or unidentified factors that, under some circumstances, can influence the final results.

## 5. Conclusions

- As expected, SLR procedures were performed faster and with less intraoperative blood loss levels, shorter postoperative stays, and fewer MAEs and SAEs than MLR procedures.
- Fewer procedures were needed to gain stabilization and repeatability in operating times and intraoperative blood loss levels in SLRs compared to MLR procedures.
- Operative time and intraoperative blood loss cannot be surrogates for SAE risk in MLRs, as they present significantly different learning curves.
- In MLR procedures, SAE reduction is gained significantly later than operative time and intraoperative blood loss level stabilization.

## Figures and Tables

**Table 1:** Baseline characteristics of patients and procedures.

	Total (n = 158)	SLRs ^%^ (n = 107)	MLRs ^%%^ (n = 51)	*p* *
Mean age (±SD ^$^) [years]	57.60 (±13.03)	58.20 (±13.89)	56.31 (±11.03)	0.407
Mean BMI ^$$^ (±SD ^$^) [kg/m^2^]	26.88 (±4.57)	26.80 (±4.61)	27.08 (±4.52)	0.731
Male (%)/Female (%)	82 (51.90%)/ 76 (48.10%)	55 (51.40%)/ 52 (49.60%)	27 (52.94%)/ 24 (47.06%)	0.856
Median operating time (IQR ^$$$^) [min.]	205 (IQR: 165)	170 (IQR: 600)	400 (IQR: 195)	<0.001 **
Median postoperative hospital stay (IQR ^$$$^) [days]	6 (IQR: 3)	5 (IQR: 3)	8 (IQR: 7)	0.016
Median intraoperative blood loss (IQR ^$$$^) [mL]	330 (IQR: 540)	170 (IQR: 155)	450 (IQR: 980)	<0.001 **
Number of minor adverse effects (%)	71 (44.94%)	46 (42.99%)	25 (49.02%)	0.476
Number of severe adverse effects (%)	28 (17.72%)	15 (14.02%)	13 (25.49%)	0.077
Incidence of Pringle’s manouver (%)	81 (51.27%)	58 (54.21%)	23 (45.10%)	0.284
Median time of Pinard’s manouver (IQR ^$$$^) [min.]	15 (IQR: 30)	15 (IQR: 30)	30 (IQR: 15)	0.534

^$^ SD-standard deviation; ^$$^ BMI-body mass index; ^$$$^ IQR-interquartile range; ^%^ SLRs-small liver resection procedures; ^%%^ MLRs-major liver resection procedures; * compared between MLRs and MLRs; ** statistically significant *p* value.

## Data Availability

The data presented in this study are available on request from the corresponding author. The data are not publicly available due to restriction.
